# Enhancing Mechanical Energy Absorption of Honeycomb and Triply Periodic Minimal Surface Lattice Structures Produced by Fused Deposition Modelling in Reusable Polymers

**DOI:** 10.3390/polym17081111

**Published:** 2025-04-19

**Authors:** Alin Bustihan, Ioan Botiz, Ricardo Branco, Rui F. Martins

**Affiliations:** 1 Department of Physics of Condensed Matter and Advanced Technologies, Faculty of Physics, Babeș-Bolyai University, 400084 Cluj-Napoca, Romania; alin.bustihan@stud.ubbcluj.ro (A.B.); ioan.botiz@ubbcluj.ro (I.B.); 2 Interdisciplinary Research Institute on Bio-Nano-Sciences, Babeș-Bolyai University, 400271 Cluj-Napoca, Romania; 3 CEMMPRE, ARISE, Centre for Mechanical Engineering, Materials and Processes, Department of Mechanical Engineering, University of Coimbra, Rua Luís Reis Santos, Pinhal de Marrocos, 3030-788 Coimbra, Portugal; ricardo.branco@dem.uc.pt; 4 UNIDEMI, Research & Development Unit for Mechanical and Industrial Engineering, Department of Mechanical and Industrial Engineering, Nova School of Science and Technology, Universidade NOVA de Lisboa, Campus de Caparica, 2829-516 Caparica, Portugal; 5 Laboratório Associado de Sistemas Inteligentes—LASI, 4800-058 Guimarães, Portugal

**Keywords:** energy absorption, honeycomb, TPMS, polymers, additive manufacturing, lattice structures

## Abstract

This study investigated the mechanical energy absorption properties of polymeric lattice structures fabricated using additive manufacturing. Existing studies have primarily focused on rigid or single-use materials, with limited attention given to flexible polymers and their behaviour under repeated compressive loading. Addressing this gap, the structures investigated in this study are manufactured using three flexible polymers—polyether block amide, thermoplastic polyurethane, and thermoplastic copolyester elastomer—to enhance the reusability performance. Two high-performance designs were analysed, namely honeycomb structures (inspired by pomelo peel and simply hexagonal arrangements) and 3D triply periodic minimal surface structure of the type FRD. The primary objective was to evaluate their energy absorption capacity and reusability using three repeated compression tests. These tests revealed that thermoplastic copolyester elastomer exhibited the highest energy absorption in initial impact conditions, but lower values for the following compressions. However, polyether block amide demonstrated superior reusability, maintaining a consistent energy absorption efficiency of 56.1% over multiple compression cycles. The study confirms that modifying triply periodic minimal surface structures along the z-axis enhances their absorption efficiency, with even-numbered z-parameter structures outperforming odd-numbered ones due to their complete cell structure. These findings highlight the critical role of structural geometry and material selection to optimise polymeric lattice structures for lightweight reusable energy absorption applications, such as automotive safety and impact protection.

## 1. Introduction

In recent years, polymeric energy absorption structures have gained significant interest in a multitude of engineering fields. Their main applications include the aerospace industry [1]; personal safety equipment, such as helmets; automotive passive safety systems; highway safety; and packaging of valuable valuables [2,3]. This growing interest has been driven by advances in additive manufacturing (AM) and simulation software, which have significantly facilitated prototyping and numerical analysis.

These technologies have not only improved the feasibility of complex designs but have also expanded the possibilities for optimising mechanical properties in energy-absorbing structures. The importance of systematic reviews in this field cannot be overstated. Comprehensive insight into the crashworthiness domain was provided by Isaac and Duddeck [4]. Their review covered models commonly used for the absorption of mechanical energy, as well as production methods using 3D printing technology, testing methods, and key characterisation parameters for energy absorption structures. Moreover, this study offers important insights into possible directions for developing existing structures and refining testing methodologies.

Other review studies, such as the one by Gandhi et al. [5], present the simulation process behind the prototyping in the case of metallic materials using lattice structures for lightweight products. A common conclusion among these studies highlights the need to incorporate fibre reinforcement in 3D printing.

However, a gap exists in the current literature regarding the optimisation of flexible polymeric lattice structures for energy absorption. While much research has explored rigid polymer and metallic structures, the potential of flexible polymers like polyether block amide (PEBA), thermoplastic polyurethane (TPU), and thermoplastic copolyester elastomer (TPC) remains underexplored. This manuscript addresses this gap by evaluating the performance and reusability of flexible polymeric lattices, specifically focusing on their energy absorption capabilities and how these materials perform under three multi-cycle compression tests.

From the point of view of mechanical energy absorption lattice structures, Helou and Kara [6] identified two main categories regarding geometric modelling: manually generated structures and mathematically generated structures. The first category, manually generated structures, includes structures such as honeycomb [7,8,9], origami structures [10,11] and beam-based lattice structures [12,13,14]. Structures such as honeycomb have been modified to enhance the mechanical properties. Several studies vary the geometry of the wall [15], while others modified the wall thickness in the compression direction [16]. Other approaches involved rotating the upper plane in phase with the lower plane at different angles to influence the failure direction [17]. Nevertheless, in general, the original form with constant wall thickness performs better or similar. The second category of mathematically generated structures use an algorithm to create the lattice. This type of structure includes triply periodic minimal surface (TPMS) lattice structures [18], which offer a superior surface-to-volume ratio and exhibit great mechanical resistance compared to beam-based lattice [1]. The study in [19] presents remarkable results for specific energy absorption (SEA), particularly for FRD structures, with 32.47 kJ/kg in quasi-static compression tests and 99.91 kJ/kg in case of dynamic tests. Furthermore, in this investigation, it was an objective to introduce a novel variant of the TPMS FRD structure in which the dimension of unit cells along the z-axis is increased to produce a configuration fully aligned with the primary direction of mechanical loading. This z-axis optimisation represents a key innovation of the study proposed to enhance energy absorption capacity in case of 3D-type structures.

In terms of mechanical properties, bioinspired structures can improve energy absorption by using a specific linear plateau. Notable examples include structures inspired by turtle shells [20], balsa wood [21], or cork wood [22]. A high SEA coefficient was achieved in studies on bio-inspired honeycomb structure based on bamboo [23] and pomelo peel [24]. The SEA coefficient is important in designing with a higher capacity while maintaining reduced weight. A good example where this is favourable is the case of the automotive or aeronautical industries, where weight reduction implies lower emissions of harmful pollutants into the atmosphere.

In many articles, researchers aim to improve the mechanical properties of structures by using specific optimisation algorithms or empirical formulas, such as the Gibson–Ashby model, to choose an appropriate lattice volume fraction [18]. In general, it is necessary to consider all parameters to improve the structure, because modifying one parameter affects others. For instance, Khan and Riccio [1] established a relationship between relative density, lattice unit cell, and volume fraction.

Furthermore, a clear design fabrication procedure should be established for additive manufacturing lattice structures considering the specificities of each 3D printer. Post-production procedures and new non-invasive testing methods are needed for a more accurate and reliable verification of simulation results. An important issue in the literature is that many results are not comparable [6]. This emphasises the need to first conduct a systematic review of lattice structures. In this way, it was possible to identify the theoretically optimal structure for absorbing mechanical energy. Additive manufacturing involves a wide variety of 3D printing methods, such as stereolithography (SLA) [25], selective laser sintering (SLS) [26], selective laser melting (SLM) [27], and multi-jet fusion (MJF) [28], among others. Fused deposition modelling (FDM), which involves extruding thermoplastic filaments, is also a commonly used process for printing polymer-based structures [2], due to its versatility and simplicity. Regarding 3D-printed polymers, the most commonly used materials include polylactic acid (PLA) [15,29,30]; thermoplastic polyurethan (TPU) [7,9,31]; nylon [32,33]; and acrylonitrile butadiene styrene (ABS) [30,34], among others. Nevertheless, data on flexible polymeric materials, such as polyether block amide (PEBA) and thermoplastic copolyester (TPC), as well as on their reusability are scarce.

This study aims to optimise the mechanical energy absorption properties of polymeric lattice structures fabricated using additive manufacturing. Two high-performance designs were selected: a 2D honeycomb-inspired configuration, and a 3D triply periodic minimal surface structure also known as FRD. Lattice structures were produced via fused deposition modelling considering three flexible polymers (PEBA, TPU 95A, and TPC) and different spatial configurations. To achieve this, an optimisation algorithm that simulates compression tests was used to iteratively adjust the main geometrical parameters to improve mechanical performance. The most efficient structures in terms of energy absorption were then fabricated and tested experimentally under compression to validate the numerical predictions. The reusability of the three selected polymeric materials was also investigated and compared.

## 2. Materials and Methods

### 2.1. Lattice Structures

After a careful literature review, several structures with superior mechanical energy absorption capacity were identified. In the first stage, all promising configurations were selected without considering their suitability for the selected additively manufactured process. The production feasibility of all these structures was assessed at a later stage. As a result, two new structure configurations were chosen for further investigation, along with a simple honeycomb structure. The honeycomb structure was maintained as a reference to effectively characterise the new materials used in this study. The newly selected structures were sourced from Zhang et al. [24] and Yin et al. [19]. These new ones presented significant differences in terms of structure topology and material features.

The first selected structure, the honeycomb, was designed to mimic the shell of a pomelo and is classified as a 2D structure. On the contrary, the second selected structure, the FRD, is a triply periodic minimal surface and belongs to the category of 3D structures. A key distinction between these structures lies in their modelling approach. The honeycomb structure was geometrically modelled using SolidWorks 2024 software [35], whereas the TPMS structure was generated using mathematical equations. For this purpose, nTop 4.1 software was used [36].

**Figure 1:**
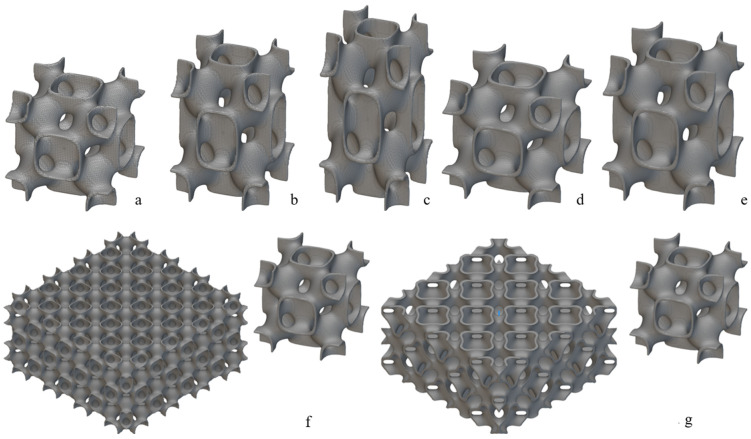
FRD TPMS lattice structures: (**a**) FRD222 base cell; (**b**) FRD223 base cell; (**c**) FRD224 base cell; (**d**) FRD333 base cell; (**e**) FRD334 base cell; (**f**) three-dimensional strucuture arragement for the FRD222 base cell; (**g**) three-dimensional strucuture arragement for the FRD333 base cell.

The honeycomb structure mimicking the pomelo peel was selected since this design demonstrated the best performance among the honeycomb-type structures examined, particularly in terms of the specific energy absorption (SEA) parameter and the overall energy absorption mechanisms. The objective was to vary the material to assess how this structure behaved when produced using three flexible polymeric materials, namely PEBA, TPC and TPU. Additionally, its reusability and energy absorption capacity within the class of polymeric materials were also studied. Regarding the TPMS structures (Figure 1), they were chosen based on their superior performance, compared to other similar structures. Similar to the honeycomb structures, the materials were varied to evaluate their reusability. Furthermore, in this case, structural parameters were also modified to enhance energy absorption performance.

The FRD TPMS structures were generated based on Equation (1) [37]:

ϕ(r) = 4 cos(X) · cos(Y) cos(Z) − [cos(2X) cos(2Y) + cos(2X) cos(2Z) + cos(2Y) cos(2Z)] = 0(1)

where *X*, *Y* and *Z* represent the spatial coordinates of the lattice base cell in terms of length, width and height, respectively. The modelling, as mentioned above, was performed using nTop software, first defining the specific general physical domain. The general size of the defined domain was 50 × 50 × 25 mm^3^. However, to fit an exact number of base cells (see Figure 1f), these dimensions were adjusted to 50.26 × 50.26 × 25.13 mm^3^. In this way, the FRD222 base cell, displayed in Figure 1a, fully met the requirements, while the dimensions remained as close as possible to the initial general size constraints. The numeric designation, such as FRD222 or FRD333, indicates the dimension proportions of base unit cells aligned along the X, Y, and Z axes, and the terms are presented in Equation 1. For instance, FRD222 indicates that the base cell has equal proportions in all directions (X = Y = Z = 2), while FRD334 defines a base cell that is geometrically stretched along the Z-axis (X = Y = 3; Z = 4), which is also the direction of compressive loading during mechanical testing. Importantly, modifying the (X, Y, Z) parameters affects the internal geometry of the unit cell without changing the external dimensions (e.g., height) of the printed sample. This approach enables a controlled investigation into how cell anisotropy along the loading axis influences energy absorption and structural response under repeated compression. It is important to note that this base cell, FRD222, has a smaller volume than the FRD333 base cell, shown in Figure 1d, although the base cell geometry is the same. Based on the hypothesis that modifying the *Z* parameter of the base cell could improve the structure’s performance as it approaches a tubular configuration—commonly used in automotive energy absorbers—different base cells were created, as exhibited in Figure 1a–e. For instance, configurations FRD223 (Figure 1b) and FRD224 (Figure 1c) are elongated along the z-axis compared to the FRD222 base cell (Figure 1a), while configuration FRD334 (Figure 1e) exhibits an elongated z-axis compared to an FRD333 base cell (Figure 1d). This wide range of cases allows us to better understand how these spatial arrangements affect the energy absorption performance.

**Figure 2:**
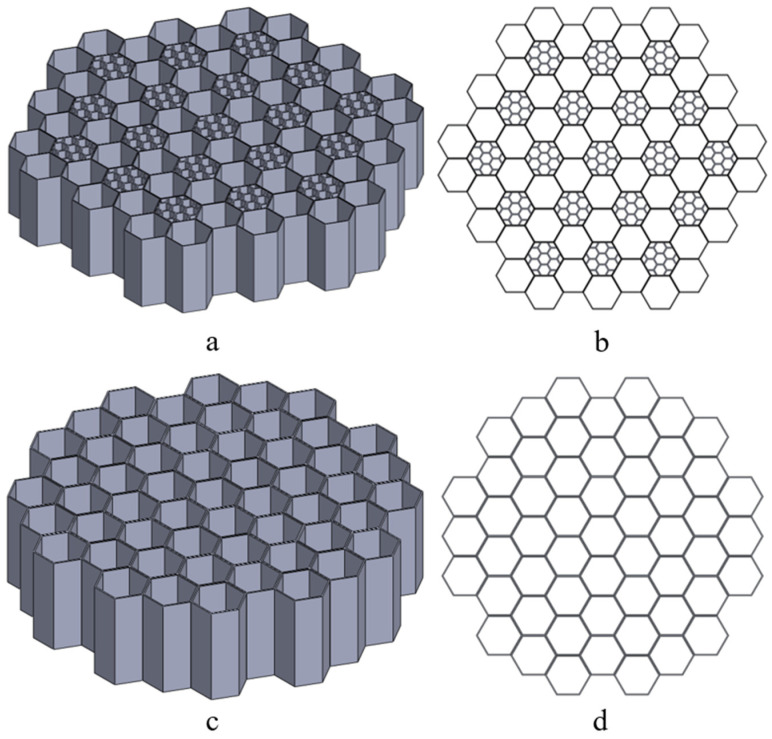
Pomelo peel-inspired honeycomb structure: (**a**) three-dimensional and (**b**) top-plane views of structure arrangement. Hexagonal structure: (**c**) three-dimensional and (**d**) top-plane views of structure arrangement.

Regarding the 2D honeycomb-inspired structures, two alternative arrangements were studied: a pomelo peel-inspired structure named H-Pomelo and a simple hexagonal structure named HEX. The former, as shown in Figure 2a, was adapted to meet the constraints of the selected 3D printing process. H-Pomelo was constructed using two types of basic cells, both with a constant wall thickness of 0.4 mm and a height of 25 mm. The length of the larger hexagon side was set to 6 mm, while the length of the smaller hexagon side was set to 2 mm. For a better understanding of the structure arrangement using these two types of base structures, Figure 2b presents a top-plane view of a sample. Concerning the simple hexagonal structure (HEX) (see Figure 2c), the base cell had a height of 20 mm, an outer hexagonal side length of 5.5 mm, and walls with a constant thickness of 0.4 mm. The goal was to establish these dimensions as a benchmark for our studies, since they have been previously used in other research conducted by the authors and have proven their efficiency compared to taller variations where the buckling phenomenon becomes more pronounced. In our case, this phenomenon also depends on the hexagon’s size (length and thickness) and its height.

### 2.2. Material Properties and 3D Printing Parameters

The materials used in this study belong to the class of flexible polymeric materials. Flexible polymeric materials were selected in order to create reusable structures. As seen in other studies that had a similar purpose [7], thermoplastic polyurethane (TPU 95A) [38] was chosen as the starting material based on its high elasticity, thermal stability during fused deposition modelling (FDM), and mechanical resilience under repeated deformation. Moreover, two other materials available in filament form that are compatible with our 3D printer and superior in terms of mechanical properties were also chosen. Therefore, it was decided to use polyether block amide (PEBA) [39] and thermoplastic copolyester (TPC) [40]. PEBA is known for its excellent fatigue resistance, low-temperature flexibility, and superior energy return, which are essential for loading scenarios such as those found in impact protection systems. TPC combines the flexibility of elastomers with the strength and processability of engineering thermoplastics, enabling it to withstand large deformations without brittle failure.

All three materials exhibit elongations at break exceeding 500%, making them ideal for reusable energy-absorbing applications [38]. Raw filament materials PEBA (Flexfill PEBA 90A by Fillamentum), TPU 95A (Ultrafuse by BASF) and TPC (Ultrafuse TPC 45D by BASF) have densities of ρ_PEBA_ = 1000 kg/m^3^, ρ_TPU_ =1149 kg/m^3^, and ρ_TPC_ =1150 kg/m^3^, respectively. Other technical specifications from the manufacturers’ data sheets were used to define the print settings and material characterisation, which are included in the references.

The printing parameters provided by the filament manufacturers were adjusted to ensure compatibility with the Bambu Lab P1S (Bambu Lab, Shenzhen Tuozhu Technology Co., Ltd., China) 3D printer used in this study, which was equipped with a 0.4 mm stainless steel nozzle. Table 1 shows the printing parameters used for each material. The TPU 95A and TPC filaments were printed using the same parameters but under different printing chamber conditions. TPU 95A was printed in an open chamber to improve ventilation, while TPC and PEBA were printed in a closed chamber to ensure better temperature stability. All energy absorption lattice structures presented in this investigation were printed with a 100% infill rate to ensure that mechanical behaviour was governed solely by the designed architecture and material properties, without internal infill variation.

### 2.3. Uniaxial Tensile and Compression Tests

**Figure 3:**
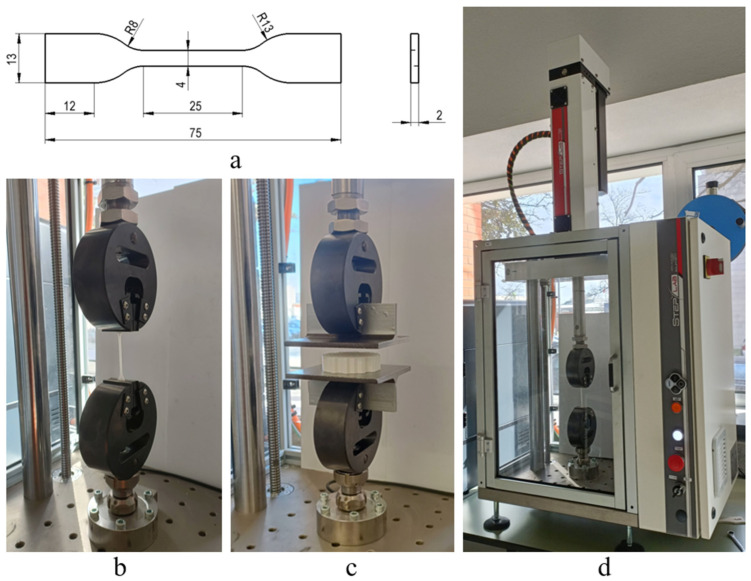
(**a**) Standard ISO 527-2:2012 [41] uniaxial tensile specimen with specific dimensions in millimetres, (**b**) standard uniaxial monotonic quasi-static tensile test set-up, (**c**) compression test set-up and (**d**) STEP Lab EA05 testing machine.

**Figure 4:**
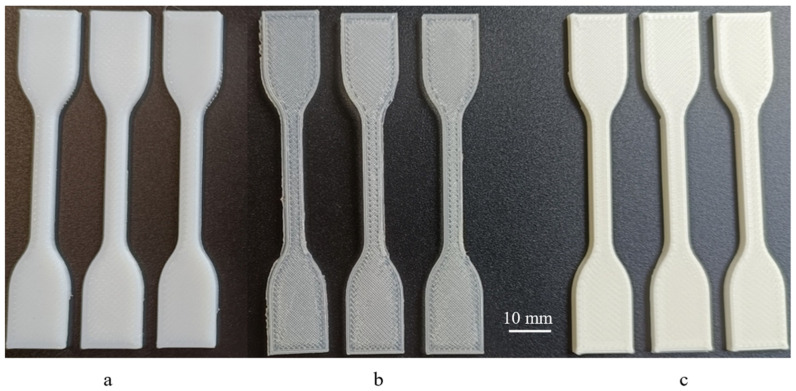
Standard ISO 527/2-5A specimens 3D-printed using (**a**) TPC, (**b**) PEBA and (**c**) TPU for tensile strength tests.

In order to characterise the mechanical behaviour of the tested materials/structures and develop robust numerical models, uniaxial quasi-static monotonic tensile tests were performed (Figure 3a,b). Tensile test specimens were produced using the printing parameters presented in Table 1. These tests were performed following the recommendations outlined in the ISO 527-2:2012 standard [41] considering the specimen dimensions presented in Figure 3a. A STEP Lab EA05 (STEP Lab S.r.l., Resana, Italy) testing machine was used, applying a constant displacement rate of 10 mm/min (Figure 3d), based on the nature of the 3 materials studied and the fact that they are highly elastic polymer types that we aim to test multiple times within an optimal timeframe to maintain their elastic state. The selected testing speed was chosen to match that used in the compression tests. This ensures a balance between these different tests. Moreover, this speed was used in another article [42] where a qualitative compression curve was observed at this testing speed. The tensile specimens tested in this study are shown in Figure 4. For each material, three tests were carried out (see Figure 3b) to determine Young’s modulus, rupture strain, and the tensile strength. Young’s modulus was defined as the slope of the initial linear elastic region of the stress–strain curve.

The FEM simulations for the FRD222 and FRD333 lattices were conducted using the values of Young’s modulus determined in the uniaxial monotonic quasi-static tensile tests. Poisson’s ratio was defined based on literature references [43,44,45,46]. The selected values were as follows: ν_PEBA_ = 0.45, ν_TPC_ = 0.48, and ν_TPU_ = 0.48. The materials were assumed to be isotropic and homogeneous. The objective of these simulations was to analyse how the energy absorption changed by modifying the z-parameter. By varying this parameter, its impact on mechanical performance—including stress distribution, deformation patterns, and overall energy dissipation efficiency—was assessed.

### 2.4. Finite Element Analysis (FEA)

FEA was conducted based on a static analysis using the nTop software to facilitate a more streamlined workflow and to leverage its capability of meshing such complex surfaces efficiently. We used a partial quad-mesh with an element size of 0.4 mm; this results in analyses with a range of finite elements between 10,634,109 and 13,473,478. The lattice structures were considered to be placed between two rigid metal plates. These plates were modelled as a metallic material with a Young’s modulus of 205 GPa, a Poisson’s ratio of 0.284, and a density of 8190 kg/m^3^. One plate was fixed, while a 500 N force was applied to the other plate. This force value was chosen to ensure that analysis remained within the initial linear zone of the compression curve, where the material exhibits a linear elastic behaviour. This approach allowed for the determination of the displacement and stress distributions experienced by the lattice structure for this loading level.

### 2.5. The 3D Printing of Lattice Structures

**Figure 5:**
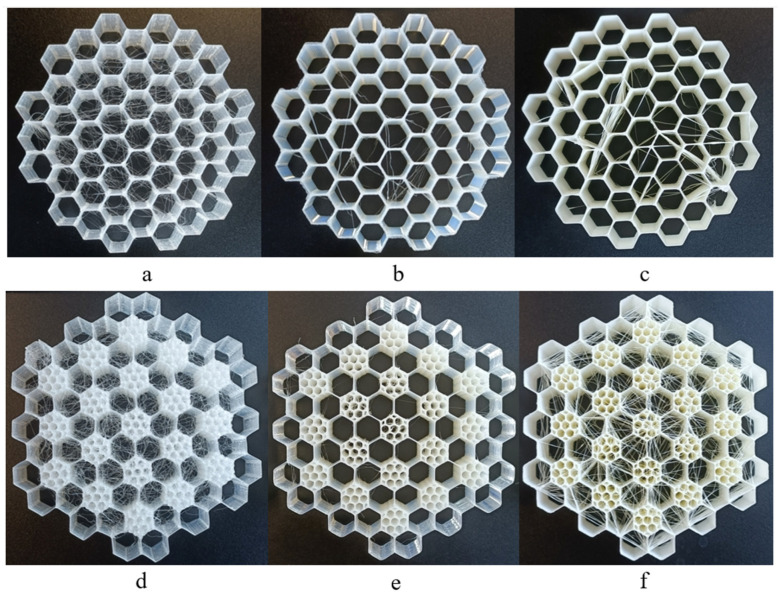
Honeycomb structures of HEX 3D-printed using (**a**) PEBA, (**b**) TPC and (**c**) TPU and HEX-Pomelo structure inspired by pomelo peel 3D-printed using (**d**) PEBA, (**e**) TPC and (**f**) TPU.

**Figure 6:**
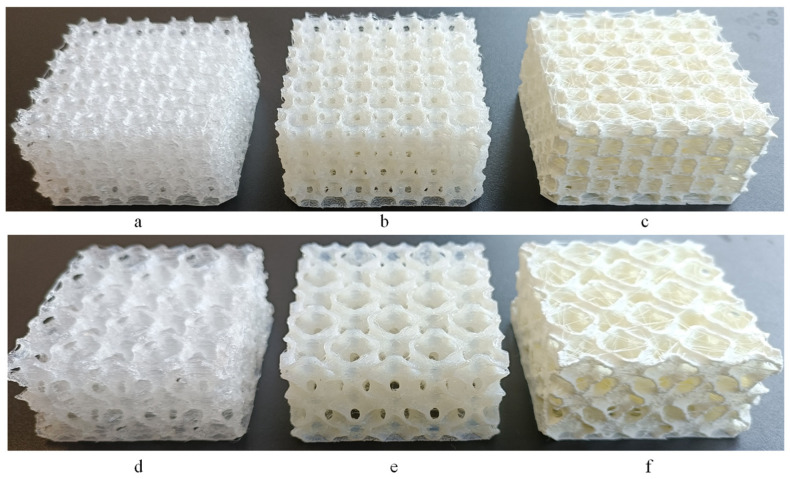
TPMS FRD222 structure 3D-printed using (**a**) PEBA, (**b**) TPC and (**c**) TPU and TPMS FRD333 structure 3D-printed using (**d**) PEBA, (**e**) TPC and (**f**) TPU.

The different types of lattice structures under investigation (Figure 5 and Figure 6) were manufactured using the same AM printer used to manufacture the uniaxial tensile specimens, and the three selected polymer materials with their printing parameters are summarised in Table 1. The lattice structures, i.e., 2D honeycomb-inspired and 3D TPMS variants, were tested under compressive loading (Figure 3c). Three compressions tests were performed on each geometry, using the same STEP Lab EA05 (STEP Lab S.r.l., Resana, Italy) testing machine, with a constant displacement rate of 10 mm/min. These tests allowed us to evaluate the mechanical performance and energy absorption capacity. In addition, they provided insights into deformation patterns and the reusability of the studied lattice structures.

### 2.6. Energy Absorption Parameters

Since this study involves two types of energy absorption structures, generally classified as 2D honeycomb and 3D TPMS lattice structures, their absorption curves exhibit distinct behaviours. Thus, it is necessary to define two methods to identify the densification point of energy in the absorption curve. For the 2D honeycomb structures, the densification point (*d_S_*) was defined as the displacement where the force reached the same value as the first maximum peak of the initial phase of the compression curve. For the 3D TPMS structures, the compression curve exhibits a continuously increasing trend, requiring a different analytical approach. To determine the densification point in the most accurate manner, relative energy absorption efficiency curves were generated, and the peak value was identified as the densification point.

The absorption capability of these structures is characterised by distinct parameters [4]. However, these parameters and their definitions may vary depending on the study. In this work, the selected parameters were absorbed energy, specific energy absorption, average compression force, compression load efficiency, and energy absorption efficiency. These selected metrics allow for a comprehensive evaluation of deformation modes, structural stiffness, and energy dissipation, which vary significantly between geometries. For the sake of clarity, these parameters are briefly defined below.

Absorbed energy (*E_abs_*) is defined as the area under the compression curve up to the densification point [47] and represents the amount of energy absorbed by the structure:

(2) $$E_{abs}=\int_{0}^{d_{s}}F\left(x\right)dx$$

where $F\left(x\right)$ denotes the applied force, and *d_s_* represents the densification point.

Specific energy absorption (*SEA*) is defined as the energy absorbed per unit of mass [48] and accounts for the ability of the structure to store energy in a unit:

(3) $$SEA=\frac{E_{abs}}{m}$$

where *m* represents the mass of the structure.

Average compression force ($F_{m}$) is defined as the ratio of energy absorbed to total compression displacement [49]:

(4) $$F_{m}=\frac{1}{d_{s}}\int_{0}^{d_{s}}F\left(x\right)dx$$

and this definition is relevant only up to the densification point; after this point, the material generally exhibits different behaviour.

Compression load efficiency (CLE) is defined as the ratio of average compression force to maximum compression force [50]:

(5) $$CLE=\frac{F_{m}}{F_{v}}$$

where $F_{v}$ is the peak of the compressive curve.

Energy absorption efficiency ($\eta_{abs}$) is used for comparative analysis of energy absorption structures and is defined as the ratio of absorbed energy to the maximum theoretical energy absorption ($F_{v}\;\cdot \;d$) [51,52]:

(6) $$\eta_{abs}=\frac{E_{abs}}{F_{v}\;\cdot \;d}$$

where *d* represents the total compression displacement up to the densification point.

## 3. Results and Discussion

### 3.1. Mechanical Properties and Analysis

**Figure 7:**
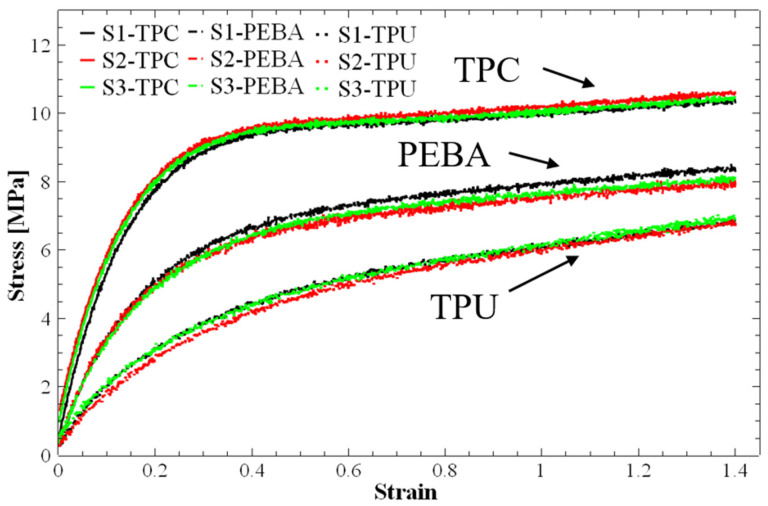
Tensile stress–strain curves obtained from standard ISO 527/2-5A additively manufactured specimens made of TPC, PEBA and TPU. S1, S2, and S3 refer to the three individual tensile specimens tested per material type to evaluate repeatability and mechanical consistency. Strain values are in [mm/mm].

Figure 7 shows the monotonic stress–strain curves obtained for the three standard specimens printed for each material. The tests were conducted under uniaxial quasi-static tensile loading, with a data acquisition rate of 4 kHz. Overall, the three curves for each material perfectly overlapped, which indicates excellent reproducibility of the tests. It is also clear that the curves extend up to a strain of 1.4. This value corresponds to the maximum distance allowed by the grip system of the testing apparatus. Thus, it was not possible to calculate the maximum elongation or rupture strain because the displacement range was not sufficient.

Based on these tests, the Young modulus (E) for each material was calculated. Their average values and the corresponding standard deviations are summarised in Table 2. These values provided essential insights into the mechanical behaviour of each material and were used in subsequent analyses, including the finite element simulation and experimental validation of energy absorption performance. Figure 7 clearly shows that TPC has the highest Young’s modulus and reaches an almost horizontal plateau first, while TPU exhibits the lowest E value and the slowest transition to fully plastic behaviour.

**Figure 8:**
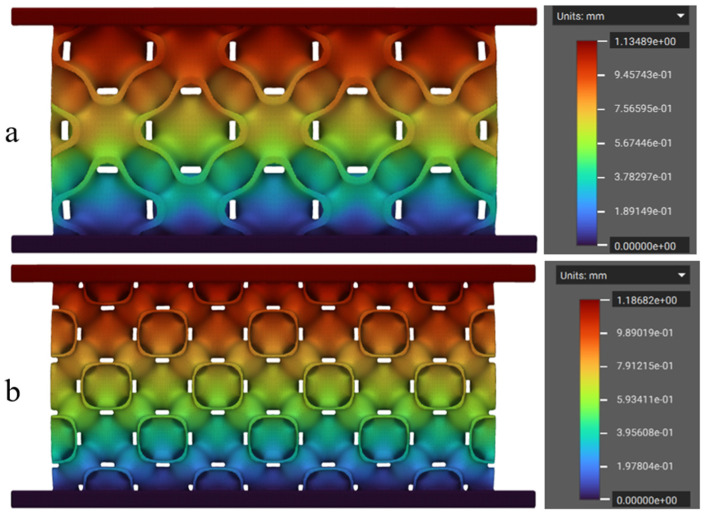
Displacement field simulated numerically for a TPMS structure made of TPC: (**a**) FRD333 and (**b**) FRD222. The pseudo-colour bars represent the displacement field.

The numerical simulations of the FRD lattice structures were performed using the values of Young’s modulus obtained from the initial linear elastic phase of the tensile stress–strain curves (Figure 7, Table 2). Poisson’s ratio values, as mentioned earlier, were taken from the literature. This analysis was initially conducted on the FRD222 and FRD333 configurations to assess differences in their theoretical mass and energy absorption behaviour. A key factor in evaluating energy absorption was the correlation between displacement and applied load. In this study, the displacement was examined under a 500 N compressive force, as this value is within the initial linear elastic region. Figure 8 shows the displacement fields simulated via the FEM for the lattice structures printed from TPC. As expected, the highest displacements were observed in the top regions of both structures, while at the bottom, due to the defined boundary conditions, displacement was zero.

It is also clear from the analysis of Figure 8 that the FRD222 configuration (Figure 8b) exhibited greater displacement of 1.186 mm, compared to the FRD333 configuration at 1.135 mm (Figure 8a). This suggests that the FRD222 architecture absorbs less energy, as it requires a larger displacement of its effective height to withstand the same applied force. In closed-cell foams, the densification point is typically defined as the moment when cell walls come into contact. Since TPMS structures tend to mimic this behaviour, a larger displacement under a lower force implies an earlier collapse of the structure, leading to reduced energy absorption. Thus, based on these findings, an architecture with greater displacement under the same load is expected to have a lower energy absorption capacity.

The next step involved the AM fabrication of four types of lattice geometries: honeycomb inspired by pomelo peel, named HEX-Pomelo; simple honeycomb, named HEX; TPMS FRD222; and TPMS FRD333, using each available material (TPU95A, PEBA, and TPC). However, a key challenge encountered in this phase was the minimum achievable resolution of the 3D printing process, particularly when using flexible polymeric materials, which are generally more difficult to print.

From a manufacturing perspective, the printing quality of the HEX and HEX-Pomelo specimens was high, as 2D structures are easier to fabricate when their growth direction aligns with the Z-axis. In contrast, for FRD structures, the printing method varied significantly depending on both the material properties and the unit cell size. Concerning material performance during printing, TPC exhibited the best visual quality, producing the most precise structures. TPU 95A generated the most printing residues, leading to a final mass higher than the theoretically calculated value. For the specimens printed using PEBA, the material exhibited good printability. However, minor structural discontinuities were observed in fine details.

Regarding TPMS printability, FRD222 structures proved to be more challenging to print than FRD333 structures due to their smaller unit cells, which led to longer printing times and a higher occurrence of structural defects. In contrast, FRD333 structures demonstrated higher print quality across all materials used, confirming the suitability of FDM printing for these structures. After printing, a preliminary mass analysis was conducted on all TPMS printed structures using manufacturer-supplied material density data and the mass estimation function in nTop software.

**Figure 9:**
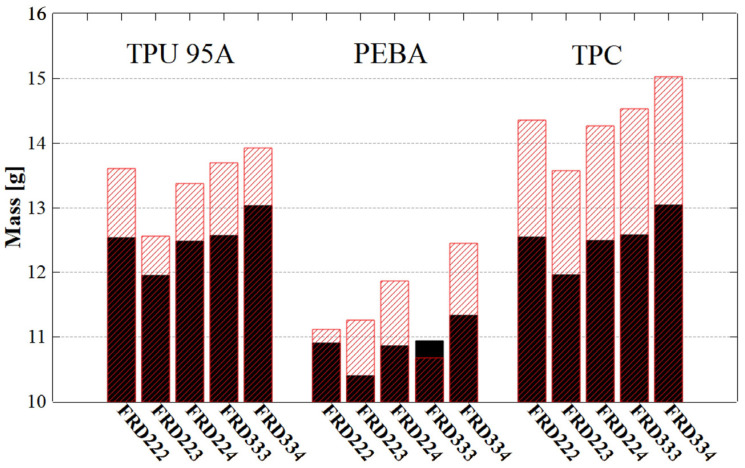
Mass analysis of TPMS structures. Black series for theoretical mass measurements and red series for experimental mass measurements.

Figure 9 compares the theoretically calculated (black series) and the experimentally measured (red series) mass values for the different geometries and tested materials. For specimens printed with TPC, even though the theoretical and experimental mass values differ, these differences remain consistent across all cases, which may indicate high print quality. It is considered that the density provided by the manufacturer may be inaccurate. To achieve results that align with theoretical values, a density value of 1322 kg/m^3^ was found to be the most accurate. For specimens printed with TPU and PEBA, more relevant variations between theoretical and experimental mass were observed. These discrepancies may be attributed to small structural defects or accumulation of residual material, which tends to occur due to nozzle travel movements during 3D printing (Figure 5 and Figure 6). Comparing the mass of different TPMS specimen configurations, those of the same type, such as FRD222 or FRD224, exhibited approximately the same mass. Regarding the FRD22z and FRD33z architectures, the mass variations remained below 1.5 g. This shows that structure variations did not directly induce significant mass differences.

**Figure 10:**
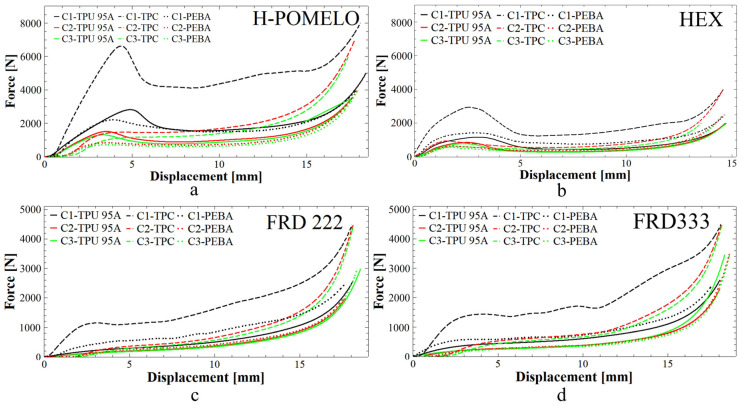
Compression curve of absorbent structures: (**a**) honeycomb design inspired by pomelo peel, (**b**) simple honeycomb design with hexagonal hole, (**c**) TPMS structures FRD222 and (**d**) FRD333. C1, C2, and C3 denote the first, second, and third compression cycles conducted on each specimen to assess recovery, reusability, and cyclic performance under repeated loading.

The compression tests for the honeycomb and TPMS structures are presented in Figure 10. These curves show the absorption response of the tested specimens in the initial phase. Each specimen was subjected to three compressive cycles identified as C1 (black), C2 (red) and C3 (green), respectively. From these graphics, it can be deduced that the specimens printed from TPC (dash lines) exhibit a higher energy absorption capacity, as indicated by the shape of the curves. This behaviour may be attributed to the higher density of this material, which is the greatest among the materials studied. It is also observed that specimens made from TPU (continuous line) and PEBA (dot lines) exhibit similar values in some cases. However, in general, PEBA shows superior energy absorption capacity.

Another significant aspect observed in the figures is the ability of the specimens to return to their original shape. For example, in Figure 10d, it can be seen that the starting points of the second (red) and third (green) curves for the FRD333 sample made from TPC are not at zero. This occurs because the arm of the compression test machine must travel a certain distance before contacting the specimen again.

**Figure 11:**
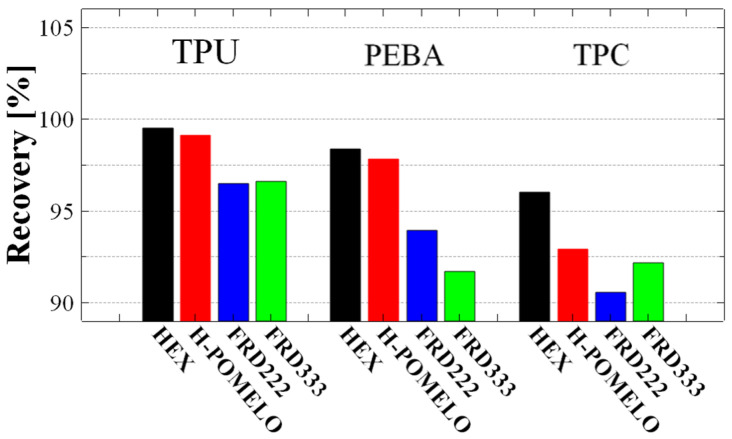
Recovery percentage of pomelo peel-inspired honeycomb (H-POMELO), simple hexagonal hole honeycomb (HEX) and TPMS absorbent structures FRD222 and FRD333 for TPU, TPC and PEBA.

By analysing this behaviour, the ability of the specimens to recover their initial shape can be quantified. This characteristic was examined after all three repeated compressions, and the recovery data obtained is presented in Figure 11. From a material standpoint, TPU exhibited the best recovery performance, reaching up to 99.5% in the case of 2D structures and 96.6% for 3D structures. These results indicate that all materials used in this study demonstrated a strong recovery capacity, with a minimum recovery of at least 90%.

This recovery capacity serves as an initial indicator of the reusability of the specimens across all cases. However, further analysis will be conducted on several key parameters that define the energy absorption performance of these structures. As previously mentioned, for 2D structures, their performance will be compared with existing literature results, where materials without recovery capability are typically used. For 3D structures, it is intended to validate the hypothesis that a smaller displacement may correlate with improved energy absorption efficiency.

**Figure 12:**
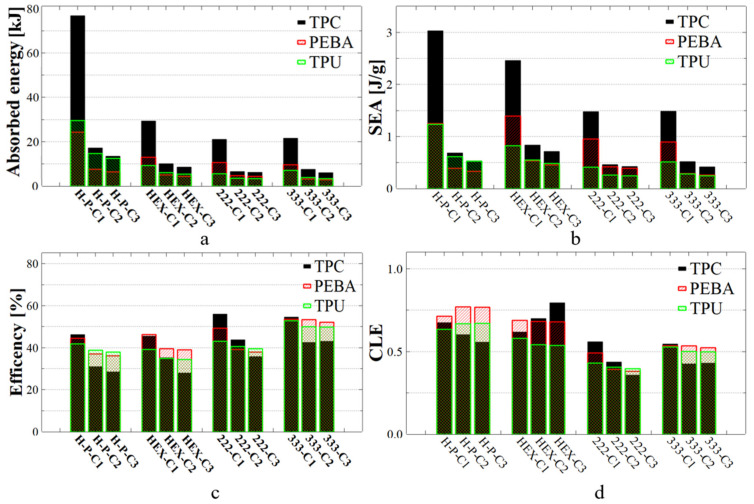
The quantity of energy absorbed (**a**), specific energy absorption (**b**), efficiency of energy absorbed (**c**) and CLE coefficient (**d**) of structures produced using TPC, PEBA and TPU. C1, C2, and C3 denote the first, second, and third compression cycles conducted on each specimen. H-P refers to pomelo peel-inspired honeycomb, HEX refers to simple hexagonal hole honeycomb, and 222 and 333 refer to FRD222 and FRD333 TPMS structures.

Figure 12a shows the absorbed energy for the tested 2D and 3D lattice structures. The H-Pomelo architecture made of TPC exhibits the highest absorption capacity (76.7 kJ) during the first compression cycle (C1). However, the absorption capacity drops significantly after the first compression, and all the specimens exhibit visible degradation. However, for the other materials, the difference between the first and second compression cycles is much smaller. In the case of PEBA, the decrease in absorbed energy was around 16 kJ, while for TPU, the reduction was around 15 kJ. After the second compression cycle, similar results were obtained for PEBA and TPU, with a more pronounced difference observed only for TPC.

Comparing the two 2D architectures, HEX specimens exhibited lower energy absorption values than H-Pomelo samples, but the differences between the three successive compression cycles were significantly smaller. The trend remains consistent, with the most substantial differences occurring in the TPC material. An interesting observation is the SEA coefficient shown in Figure 12b, where the HEX specimen made from PEBA demonstrates a superior value compared to the H-Pomelo structure made of TPU or PEBA. In the case of the TPC material, the coefficient remains similar irrespective of difference in height or geometry. Based on this observation, it can be concluded that for these types of polymeric materials, 2D structures such as H-Pomelo do not provide a significantly higher energy absorption capacity, despite their much more complex geometry compared to the HEX structure.

In the case of the 3D TPMS architectures, although they do not exhibit an increased energy absorption capacity (see Figure 12a), it can be observed that the difference between the first and second compression cycle is smaller than for the 2D structures. Additionally, for the second and third compression cycles, the variations in absorbed energy were almost similar. This characteristic suggests that these types of structures possess strong reusability potential. Regarding the SEA coefficient (see Figure 12b), TPMS architectures show reasonable values compared to the other structures studied. It is important to note that the highest SEA coefficient values were achieved for the TPC material (3.03 J/g). For PEBA, all SEA values ranged between 0.9 and 1.39 J/g. This indicates that for the lightest material used in our study (PEBA), which we believe has the best reusability characteristics, the SEA coefficient does not stand out significantly. In contrast, for TPU, the SEA coefficient is relatively low, exceeding 1 J/g only in the case of the H-Pomelo structure.

From the perspective of energy absorption efficiency, Figure 12c shows that TPMS structures exhibited higher efficiency compared to basic structures H-Pomelo and Hex. Among the 2D structures, the highest efficiency value was achieved by the H-Pomelo specimen made from TPC at 46.20%. However, this value is very close to that of the HEX specimen made from PEBA, which reached 46.09%. This finding indicates that, in terms of efficiency, the H-Pomelo samples did not exhibit significantly higher values compared to the Hex specimens. Regarding reusability, it was observed that all specimens showed similar values for the C2 and C3 compression cycles, with differences in efficiency appearing only during the first compression. The highest efficiency in terms of reusability was found for the FRD333 architecture produced from PEBA, while the weakest performance was observed for all specimens made from TPC. Although this material demonstrated the best results in the first compression cycle for three out of four structures, its performance significantly deteriorated in the subsequent two compression cycles. This trend was consistent across all studied parameters.

The CLE parameter, presented in Figure 12d, exhibited higher values in the case of 2D specimens. This fact can be explained by the larger amount of energy absorbed by these structures compared to 3D shapes. Overall, the FRD333 specimen maintained the best performance after three consecutive compression cycles. For 2D structures, an increase in CLE values was observed during the second and third compression cycles in certain cases, such as HEX structure made from TPC or HEX-Pomelo structure made from PEBA. This behaviour can be explained by analysing the shape of the absorption curve. As shown in Figure 10b, in the case of TPC, after the first compression, the curve no longer exhibits a well-defined maximum peak in its initial phase. This affects the calculation of the densification point, leading to a lower value of displacement and causing the average force value to be closer to the maximum peak value. As demonstrated in Equation (4), the average force is strongly correlated with the densification point. A similar trend was observed in the Hex-Pomelo structure made from PEBA, where the absorption curve exhibits a longer linear region, indicating a more gradual energy absorption process. However, despite these variations in the CLE values, the total amount of energy absorbed in these two structures remains significantly lower compared to the other cases.

According to the data presented in Figure 12b, the SEA values obtained for the H-Pomelo structure were lower compared to those reported in other studies [24]. In the present study, the maximum SEA value reached approximately 3 J/g, whereas in the reference study, SEA reached around 14 J/g. From the perspective of the CLE coefficient, which is a key indicator of the structure’s absorption behaviour, higher values were observed in all tested cases. Moreover, these values remained stable or even increased during the second and third compression cycles, emphasising the reusability potential of the structures designed here. This characteristic was particularly notable in lattice structures fabricated from PEBA and TPU. It is important to highlight that, in this study, polymeric materials with higher density were used compared to the metallic materials used in the reference study, and the manufacturing methods were also entirely different, which may have contributed to the observed discrepancies in SEA values.

For the HEX samples, it was possible to conclude that the absorbed energy value of 29.3 kJ for the TPC structure was significantly higher than the value reported in the reference study; see Figure 12a. In that study, the average absorbed energy was 13.2 kJ, a value comparable to the energy absorption capacity of PEBA observed in the present study. Since PEBA has a lower density than TPU 95A, its SEA coefficient, as shown in Figure 12b, exhibited higher values compared to those of the reference study. For instance, in the present investigation, for HEX structures made from PEBA, the SEA coefficient reached 1.4 J/g, while in the reference study, the average value for the best material, TPU 95A, was below 1 J/g. Conversely, TPU 95A used in the current study led to SEA values lower than those reported for the same material used in the reference study. This finding further highlights the importance of selecting high-quality materials to achieve superior structural performance.

Regarding structure efficiency (see Figure 12c), the results registered for PEBA (46.1%) and TPC (45.5%) are higher than the average value reported in the reference study (44%). This clearly indicates that TPC and PEBA exhibit superior capabilities for manufacturing high-performance shock-absorbing structures compared to the more commonly used TPU 95A. Moreover, the assumption proposed in Figure 8—that the displacement measurable in simulation is directly correlated with the absorbed energy—is clearly confirmed in Figure 12a, where the FRD222 structure absorbs less energy than the FRD333 structure. In this study, TPC was selected for numerical simulations because the lattice structures printed with this material exhibited the highest quality among the AM specimens. Based on this confirmation, new unit cells with modifications in the Z-axis parameter (FRD22z and FRD33z) were developed, more specifically FRD223, FRD224, and FRD334 architectures, as shown in Figure 1b,c and e, respectively.

### 3.2. Redesign of the Unit Cells

**Figure 13:**
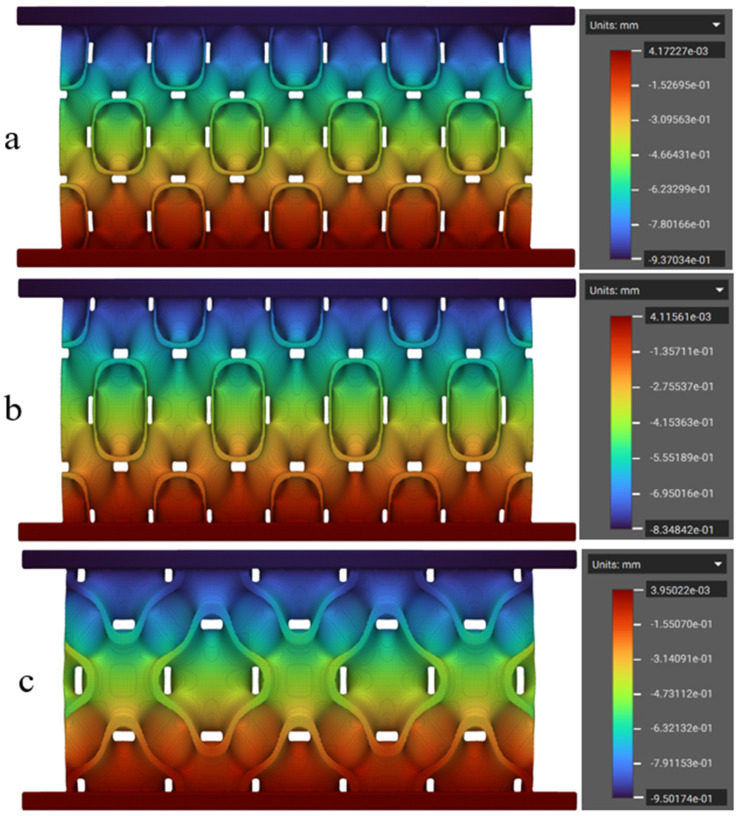
Finite element simulation of TPMS structures made of TPC: (**a**) FRD223, (**b**) FRD224, and (**c**) FRD334. The pseudo-colour bars represent the displacement field.

The newly developed unit cells—FRD223, FRD224 and FRD334—were numerically simulated using the approach previously described. The displacement fields obtained from the FEM analysis are presented in Figure 13. The results indicate that these new architectures displayed smaller displacement values compared to the reference FRD222 and FRD333 structures (Figure 8). As shown in Figure 13b, the FRD224 structure exhibited the lowest total displacement among the tested geometries. To further investigate this behaviour, the structures were printed via FDM using the three selected materials and subsequently tested under compression, following the same procedure as in the previous experiments.

**Figure 14:**
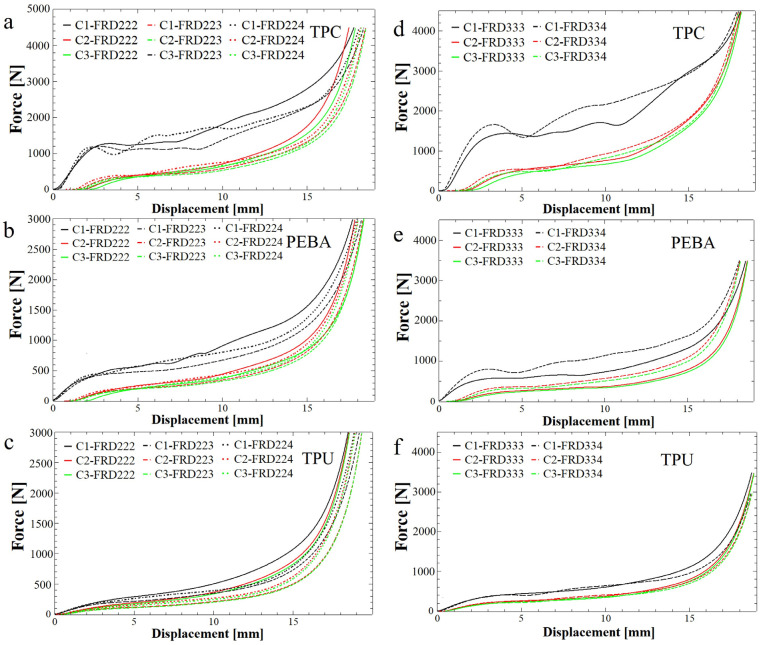
Compression curves of FRD22z-type structures produced using (**a**) TPC, (**b**) PEBA and (**c**) TPU and FRD33z-type structures produced using (**d**) TPC, (**e**) PEBA and (**f**) TPU. C1, C2, and C3 denote the first, second, and third compression cycles conducted on each specimen to assess recovery, reusability, and performance under repeated loading.

Figure 14, left side, shows the absorption curves for the FRD223 and FRD224 structures as well as for the reference FRD222 structure. The most notable aspect of the newly proposed structures is the increased length of the absorption curve, which indicates a greater distance to the densification point and, consequently, a higher amount of absorbed energy. This observation is consistent with the geometric modifications introduced in the FRD223 and FRD224 structures, where the distance between the walls of a unit cell along the z-direction is greater compared to the reference FRD222 structure. Thus, a larger displacement is required before the walls come into contact. The longer distance to the densification point and the higher absorbed energy may also be correlated with the smaller z-axis displacements observed in the simulation presented in Figure 13a,b.

Figure 14 shows an overlap or crossing of the compression curves, particularly in later cycles. This behaviour is attributed to subtle variations in the elastic recovery rate and local deformation across individual samples and layers. These effects may arise from differences in unit cell completeness (especially in odd z-axis structures), minor inconsistencies in 3D printing, or progressive material softening during compressive loading tests.

Regarding the FRD334 structure and its reference FRD333 structure, their absorption curves are displayed in Figure 14, right side. By comparing the results, it can be concluded that the absorption curves exhibit similar lengths, which is expected because FRD33z has more similar Z-proportions (1.333 for FRD334) compared to the FRD22z structures (1.5 for FRD223 or 2 for FRD224). Nevertheless, in this case, as observed in Figure 14d or Figure 14e, the area under the absorption curve appears larger for the FRD334 structure, indicating an increase in absorbed energy. This can be explained by the smaller displacement along the z-axis compared to the FRD333 structure (Figure 8a), as shown in the simulation in Figure 13c.

**Figure 15:**
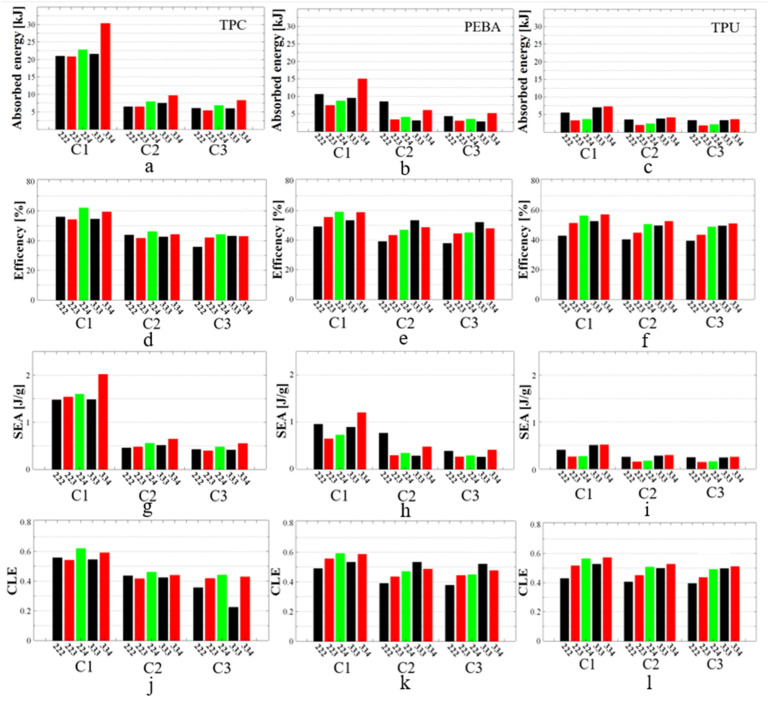
Specific values of TPMS FRD structures for energy absorbed in case of (**a**) TPC, (**b**) PEBA and (**c**) TPU; energy absorption efficiency in case of (**d**) TPC, (**e**) PEBA and (**f**) TPU; specific energy absorption in case of (**g**) TPC, (**h**) PEBA and (**i**) TPU; and crushing load efficiency in case of (**j**) TPC, (**k**) PEBA and (**l**) TPU. C1, C2, and C3 denote the first, second, and third compression cycles conducted on each specimen.

Figure 15a–c show the results of energy absorption for all tested FRD structures fabricated from the three selected polymers. Among the studied structures, FRD334 exhibited the highest values across all materials. The maximum absorbed energy, measured at 30.3 kJ, was observed for the TPC material (Figure 15a) and exceeded the value obtained for the 2D honeycomb HEX structure. According to the numerical simulations presented in Figure 13, FRD223 and FRD224 structures were expected to demonstrate higher energy absorption than the FRD334 structure, based on their smaller z-axis displacements. However, it was observed that the FRD334 structure was followed by different structures depending on the material used. As shown in Figure 15b, for PEBA, the second highest energy absorption value is observed in the FRD222 structure, while for TPU (Figure 15c), it was for the FRD333 structure.

From a structural perspective, the most relevant results are those obtained with TPC (Figure 15a), as the quality of the printed samples is the best for this material. This means that the physical structure is the closest to its FEM structure. Differences in manufacturing quality are influenced by the level of structural detail. A notable example is the FRD224 structure, which, based on simulations, was expected to exhibit a higher absorption capacity than the FRD334 structure. This discrepancy is attributed to the lower printing quality of structures with a cell size of two, as these designs include finer details and bridging gaps, which are challenging to print accurately. From both a visual and an energy absorption standpoint, FRD333 and FRD334 architectures are better suited for production using FDM technology. Although FRD222 structures can also be manufactured, achieving optimal quality would require a more in-depth investigation of printing parameters and physical constraints. For instance, using a smaller nozzle size could improve accuracy.

Regarding energy absorption efficiency, as shown in Figure 15d, the most efficient structure during the first compression cycle was the FRD224 structure produced from TPC (62%). However, its efficiency decreased after the second compression cycle to 46.1%. To identify the most stable structure in terms of energy absorption efficiency, Figure 15e shows that FRD333, produced from PEBA, maintains the best performance, with 56.1% after the first compression cycle and 52.1% after three consecutive compression cycles. A similar trend was also observed in the FRD334 structure produced by PEBA or TPU. In this case, the results of the second and third compression cycles were close to those observed in the initial compression cycle (Figure 15e,f). A comparable behaviour occurred in the FRD224 structure (Figure 15e,f), but the difference between the first and second compression cycles was greater than in the case of the FRD334 structure.

The performance evaluation revealed that PEBA exhibited the highest reusability, maintaining energy absorption efficiency of 56.1% even after three successive compression cycles. This consistency suggests minimal degradation, making PEBA a strong candidate for applications requiring repeated impacts. In contrast, TPC showed high initial absorption (e.g., 76.7 kJ for H-Pomelo and 30.3 kJ for FRD334) but poor retention across compression tests, with efficiency declining below 50% by the third compression cycle. This trade-off is likely due to TPC’s higher stiffness and lower elastic recovery, which lead to permanent deformation under repeated loading.

Specific energy absorption (SEA) analysis, as presented in Figure 15g–i, indicates that the FRD334 structure exhibits the best results across all tested materials, with the highest value of 2.02 J/g for TPC. For the remaining samples, the trends established from the energy absorption histograms (see Figure 15a–c) remain consistent because the specimen weights are comparable, with the differences between specimens made from the same material being less than 2 g, as shown in Figure 9. In addition, based on these features, the lighter structures such as FRD223 or FRD333 are advantageous in terms of mass compared to FRD222 or FRD334 but without any improvement in the SEA coefficient. A clear example of this behaviour is displayed Figure 15h for PEBA.

This phenomenon can be explained by the fact that structures such as FRD222 or FRD334, with an even z-axis number, contain complete sets of base cells, which contribute to energy absorption in the entire structure. Conversely, structures with an odd z-axis number include only a fraction of the final row of cells, meaning that a portion of the structure remains incomplete, and energy absorption is not fully achieved by a complete cell geometry. For better understanding, Figure 1g,f may be consulted, as they present the differences between the upper planes of the FRD333 structure (with an odd z-axis configuration) and the FRD222 structure (with an even z-axis configuration). In summary, although partial structures such as FRD223 have lower weight, their energy absorption capacity is reduced compared to structures such as FRD224, because the absorption process is compromised by an incomplete cell geometry of the last layer of the structure.

The analysis of the CLE coefficient (see Figure 15j–l) also shows the superior performance of the FRD224 and FRD334 structures. It is clear from the figure that these structures are the most efficient across all tested materials. This observation can be explained by the same fact that structures such as FRD222 and FRD334, with an even z-axis number, contain complete sets of base cells which contribute to energy absorption in the entire structure.

CLE values were generally above 0.45 after the three consecutive compression cycles. This value is not exceptionally high, primarily because the selected densification point was based on the energy efficiency curve. Other studies have often defined the densification point based on visual observation, with this point being identified as the moment when the structure’s walls begin to come into contact. This approach generally yields better theoretical results for the CLE in most cases because the absorption curve exhibits a shorter but linear region. However, our method was selected because it is considered the most practical and accurate way to determine the densification point. Additionally, the maximum force value (F_v_) from Equation (5) was adopted as the force encountered at the densification point. This approach also contributed to the negative CLE coefficient values.

Nevertheless, the primary goal of the present study was to demonstrate the differences among TPMS structures of the FRD type. Thus, it has been shown that, in all the presented cases, the FRD334 structure exhibits the most favourable values for the parameters related to mechanical energy absorption. Furthermore, this structure, which has been modified along the z-axis, consistently demonstrated superior performance compared to the reference FRD333 structure. Therefore, it can be concluded that modifications along the z-axis improve the structure’s absorption capacity, particularly when the z-parameter coincides with the compression testing axis of the specimens. It is also recommended that this parameter takes even values to ensure the formation of a structure with the maximum number of complete unit cells. From a material standpoint, TPC demonstrates the highest energy absorption capacity during the first compression, whereas PEBA is considered the most reusable material in terms of efficiency and durability, exhibiting stronger long-term performance characteristics. The performance of TPC, characterised by high initial absorption followed by sharp deterioration, can be explained by its high Young’s modulus and the row material density, which contribute to excellent stiffness and energy dissipation during the first compression cycle. However, its limited strain recovery leads to irreversible plastic deformation and microstructural breakdown, impairing performance in subsequent cycles. This behaviour contrasts sharply with PEBA, which has lower initial stiffness but superior resilience and energy recovery.

### 3.3. Engineering Applications

**Figure 16:**
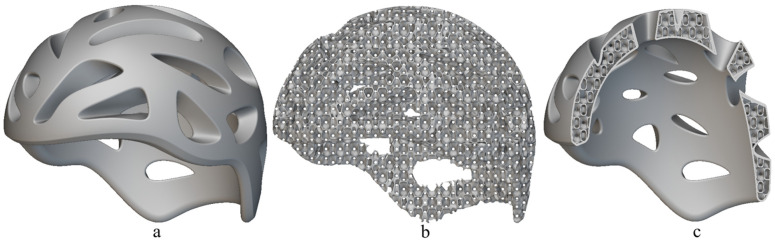
Finite element model of a safety bike helmet modelled using (**a**) outer shell and (**b**) internal FRD334 structure and both presented together in (**c**) the cross section of a bike helmet.

**Figure 17:**
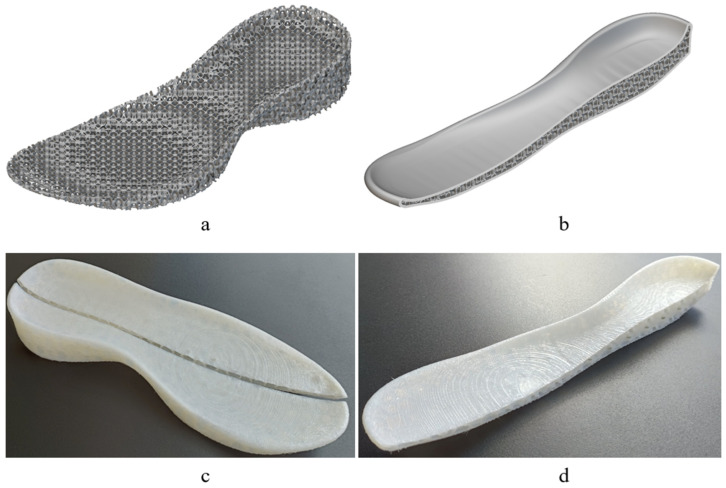
Finite element model of a shoe sole with (**a**) the internal FRD structure, (**b**) cross section of the shoes sole with outer shell, (**c**) 3D-printed sample of a shoe sole with internal FRD334 structure, and (**d**) cross section of the shoe sole with outer shell.

In order to clarify the practical applicability of these structures, two easily achievable applications using the 3D printing technology are presented. Specifically, Figure 16 and Figure 17 showcase a 3D model of a safety bike helmet and a 3D model along with the final product of a shoe sole, respectively. The base model of the protective helmet was sourced from the GrabCAD community library [53] and, subsequently, the internal structure was replaced with the FRD334 architecture, as shown in Figure 16b. An outer shell, presented in Figure 16a, was retained to bond the incomplete structures together. A sectional view of the final helmet finite element model is provided in Figure 16c.

A similar approach was applied to the shoe sole. The base model was taken from the nTop library [54]. Figure 17a displays the internal structure of the sole. In this case, it is considered that the sole can be used without an outer shell, but the base cell size needs to be reduced relative to that of the FRD334 structure. The idea is to maintain the proportions of the FRD334 structure. Thus, we propose an FRD architecture with X, Y and Z parameters of 1.5, 1.5, and 2 to maintain the proportions found for FRD334 structure. A sectional view of the sole, which shows the embedded FRD structure, can be seen Figure 17b. Finaly, Figure 17c,d present the final product manufactured using the same 3D printer. It can be observed that the product quality is high, indicating that this type of application is highly feasible for future developments.

To contextualise the performance of the structures developed in this study, a comparison with conventional materials used in helmet liners and footwear components is relevant. Expanded polystyrene (EPS) is widely used in helmet applications due to its high specific energy absorption (SEA) and low density. However, it is a single-impact material that permanently deforms and must be replaced after a single use. In contrast, the PEBA-based FRD334 lattice presented in this work demonstrated significant advantages in terms of reusability and mechanical recovery. While the peak SEA obtained under quasi-static compressive testing reached 2.02 J/g, this value is comparable to EPS reported under high-speed impact tests. Also, the structure tested in this study maintained over 56% energy absorption efficiency after three consecutive compressive cycles compared to a 36–40% energy absorption efficiency in case of EPS-60 [55,56]. Additionally, it preserved its shape and mechanical integrity, which is not possible with EPS.

It is important to note that EPS performance is typically assessed under high-speed dynamic impact conditions, whereas the values reported in this study were obtained under quasi-static compression tests. The difference in strain rate and deformation mechanisms must be taken into account when interpreting SEA values.

Similarly, in sports and running footwear, thermoplastic polyurethane (TPU) is widely used for its energy return and cushioning properties. However, such systems often lack effective energy dissipation, especially under repeated impact. In contrast, the PEBA-based FRD334 structure presented in this study demonstrated high elastic recovery (~92%) after three compression cycles, indicating its suitability for dynamic applications where both comfort and protection are essential.

Nevertheless, the ability of PEBA-based 3D-printed lattices to withstand repeated impacts while offering comparable energy absorption levels highlights their potential for next-generation protective systems, particularly in applications where multi-impact resistance, comfort, and long-term durability are required, such as advanced helmets and performance footwear.

## 4. Conclusions

This study explored the energy absorption capabilities of additively manufactured polymeric lattice structures produced by fused deposition modelling. The studied structures included 2D structures, namely honeycomb inspired by pomelo peel and simple hexagonal patterns and 3D triply periodic minimal surface (TPMS) structures with different cell unit architectures (FRD222, FRD223, FRD224, FRD333, and FRD334). Three polymeric materials—polyether block amide (PEBA), thermoplastic polyurethane (TPU), and thermoplastic copolyester (TPC)—were used. The following conclusions can be drawn:

- The tests carried out on these structures were designed to evaluate their ability to withstand repetitive compressive forces while maintaining structural integrity and reusability. It was demonstrated that material selection played a crucial role in both energy absorption capacity and reusability.
- TPC exhibited the highest initial energy absorption values, with 76.7 kJ observed for the Hex-Pomelo honeycomb structure and 30.3 kJ for the FRD334 TPMS structure. However, a significant degradation in material performance was observed after three repeated compression cycles, indicating a lower reusability potential.
- In contrast, PEBA, although initially absorbing slightly less energy initially, maintained its absorption efficiency over multiple cycles. Specifically, the FRD334 structure made from PEBA retained an energy absorption efficiency of 56.1% after three compressions, making it the most viable candidate for applications requiring reusability.
- Modifying the z-axis parameter in TPMS structures can significantly enhance their energy absorption capabilities. The FRD334 structure, designed with an even-numbered z-axis configuration, consistently outperformed other TPMS structures, including FRD222 and FRD223, in terms of energy absorption and SEA coefficient. The highest SEA coefficient for a 3D structure was recorded for FRD334 made of TPC (2.02 J/g).
- Even-numbered z-axis structures are more effective in terms of energy absorption performance because they allow for complete unit cell formation, thereby ensuring uniform energy distribution during compression. In contrast, odd-numbered z-axis structures incorporate an incomplete final cell row, resulting in diminished absorption performance.
- From a manufacturing perspective, it was revealed that the precision of the 3D printing process affected the mechanical behaviour of energy-absorbing structures. TPC showed superior print quality while TPU demonstrated the lowest print quality due to excessive mass accumulation from residual printing material.
- Two-dimensional honeycomb structures exhibited high initial energy absorption values but degraded more rapidly after repeated compressions. In contrast, TPMS structures, particularly the FRD334 architecture, demonstrated greater consistency in their performance across multiple compressions, highlighting their suitability for reusable energy absorption systems.
- The energy absorption efficiency remained stable in PEBA-based structures, with minimal degradation between compression cycles, supporting their potential use in safety equipment, automotive crash components, and aerospace impact protection systems. This consistent performance highlights their suitability for environments where repeatability is critical.

This research provides important insights into the optimisation of polymeric energy-absorbing structures, with potential applications in automotive crash safety, sport or shoe industry and personal protective equipment. The findings suggest that future design efforts should focus on refining z-axis modifications in TPMS structures to further enhance SEA values and overall energy absorption efficiency.

Furthermore, it was confirmed that the choice of material, structural geometry, and manufacturing process are all critical factors in designing effective energy absorption structures. Among the evaluated designs, the FRD334 structure, particularly when fabricated using PEBA, exhibited the best balance between energy absorption, efficiency, and long-term reusability, representing a promising combination for next-generation impact protection solutions.

## Figures and Tables

**Table 1:** Printing parameters used for energy absorption structures.

Parameter	TPU 95A	TPC	PEBA
Printing Speed	30 mm/s	30 mm/s	30 mm/s
Max. Acceleration	300 mm/s^2^	300 mm/s^2^	300 mm/s^2^
Nozzle Temperature	230 °C	230 °C	240 °C
Platform Temperature	35 °C	35 °C	75 °C
Fan Speed	100%	100%	50%

**Table 2:** Young’s modulus for the tested polymer produced by fused deposition modelling.

E_PEBA_ (MPa)	E_TPC_ (MPa)	E_TPU_ (MPa)
32.62 ± 0.97	49.69 ± 0.7	14.49 ± 0.9

## Data Availability

Data will be made available on request.
